# Supplementary material for: A common pathway controls cell size in the sepal and leaf epidermis leading to a nonrandom pattern of giant cells
Source: PLoS Biol. 2025 Nov 3;23(11):e3003469. doi: 10.1371/journal.pbio.3003469 (PMC12599956; doi:10.1371/journal.pbio.3003469)
Supplement: S1 Table — (PDF) [file pbio.3003469.s023.pdf]

**Genotype**	Primer 1 (5′ to 3′)	Primer 2 (5′ to 3′)	Primer 3 (5′ to 3′)
acr4-2	TAGTCACTCTGTGGAATGTCTC (WT) GCACCTACAATTCCTCAATCTG (mut)	GCACCTACAATTCCTCAATCTG (WT) GCCTTTTCAGAAATGGATAAATAGCCT (mut)	
atml1-3	CAGGCAGAAGAAAATCGAGAT (WT) GAAACCAGTGTGGCTATTGTT (mut)	GAAACCAGTGTGGCTATTGTT (WT) TTGGGTGATGGTTCACGTAGTGGG (mut)	
dek1-4	TCCACAGGTAGTTTCTCTTGC	TGAAGACTGAAAGGACAAAAGGTGC	
lgo-2	CTTCTCAACCTCTCACTTCTCCAA	CCGAACACCAACAGATAATT	TTGGGTGATGGTTCACGTAGTGGG
ATML1-OX	GAAACCAGTGTGGCTATTGTT	CATATGGGAGACAGCTTTCTCATACGCG	
LGO-OX	CTTCCCTTTCTCCTAAGTTCCT	GATTATCTCACAAGTCGACAC	

S1 Table. Primers for genotyping mutants and overexpression lines.
