# Supplementary material for: A common pathway controls cell size in the sepal and leaf epidermis leading to a nonrandom pattern of giant cells
Source: PLoS Biol. 2025 Nov 3;23(11):e3003469. doi: 10.1371/journal.pbio.3003469 (PMC12599956; doi:10.1371/journal.pbio.3003469)
Supplement: S2 Table — Parameters used for the simulations shown in Figs 7 and S20. All units are arbitrary. (PDF) [file pbio.3003469.s024.pdf]

Parameter	Description	Values
P_A	ATML1 basal production rate	1.19
V_A	ATML1 auto-induction rate	1.25
K_A	ATML1 concentration for half ATML1 auto-induction maximal rate	1.9
n_A	Hill coefficient for ATML1 auto-induction	5
G_A	ATML1 degradation rate	1
V_T	Target maximal production rate	10
K_T	ATML1 concentration for half ATML1-mediated target maximal production rate	2.0
n_T	Hill coefficient for ATML1-mediated target induction	1
G_T	Target degradation rate	10
Θ_T	Target threshold for inhibiting mitosis	0.6
$\Theta_{C,S}$	Timer threshold for synthesis	2
$\Theta_{C,D}$	Timer threshold for timer resetting	3
P_C	Timer basal production rate	0.1
E_0	Characteristic effective volume	15
	Exponential radial growth rate	0.007
	Exponential added growth rate to the vertical direction	0.012

S2 Table. Parameter values used for the simulations. Parameters used for the simulations shown in Figs 7 and S20. All units are arbitrary.
