# Supplementary material for: A common pathway controls cell size in the sepal and leaf epidermis leading to a nonrandom pattern of giant cells
Source: PLoS Biol. 2025 Nov 3;23(11):e3003469. doi: 10.1371/journal.pbio.3003469 (PMC12599956; doi:10.1371/journal.pbio.3003469)
Supplement: Attachment — Submitted filename: plos-biol.pdf [file pbio.3003469.s025.pdf]

This is a detailed survey of an interesting and deep aspect of plant biology – the control of physical properties of cell size and positioning. Some beautiful microscopy and comprehensive mutant analysis paints a picture connecting the same circuitry to cell size control in two different cases. Computational and modelling theory is developed to explore evidence for cell properties departing from random null models, and to explore a putative explanatory mechanism for the links observed.

I enjoyed reading this paper and think its survey of physical properties and their genetic controllers is an interesting story. I have some comments about the presentation and some concerns about the randomization-based null models (and hence the results downstream of these), but these may well be due to my own misunderstanding and I'd be very open to being corrected. The most important scientific comments are in bold as "**randomisation method point 1 & 2**" below.

For transparency, I am Iain Johnston and I am happy for this review to be treated as public domain. To my eyes my most important shortcomings as a reviewer are a lack of familiarity with the genetic players involved in the regulatory circuit under study.

Presentation comments

This is a huge paper and as such it is a challenge to read. That's fine – readers should be challenged. But the challenge is compounded by the placement and styling of important information. With this many figures, the reader could be helped substantially more.
We are many times directed to multiple subfigures of different figures to support a scientific point (e.g. "the size of the largest cells is moderately reduced in *acr4-2* mutants (Figs. 1B, 4B, 4H–J, 5B, 5H–I, 216 K), and more greatly reduced in *atml1-3* mutants (Figs. 1C, 4C, 4H–J, 5C, 5H–I, K).", l215-6; also l222, 250, 255, 269, 272, 278, 298, perhaps some more). I'm sure I'm not alone in finding it challenging to make comparisons across multiple subfigures across multiple pages.

First – parts 4HIJ and 5HIK are the same data many times over and the best way of plotting these data is actually none of these options – they should be visualised as individual datapoints, with their profile expanded to reflect density (e.g. with ggbeeswarm), with the cell area scale on a logarithmic axis. This is also the case for 3D, 2E, 2F, and any other distributions of cell area in the SI.

Second – to my eyes the most important message from these comparisons is the similarities (or differences) in the impact of the different mutants across the tissue types (sepal, early leaves, mature leaves). This could be done in one frame. Fig 5L is the best current example, but I'm confused about the normalisation (see below). Why not have pointwise, log-scaled, cell area distributions in a trellis plot for mutants (columns) and tissues (row)? Then the reader can scan across the trellis, see the similar patterns across tissues from the different genetic perturbations, and get all the information together.

Some suggestions for paring back content:

Fig 2GH don't report much (anything?) more than their correlation coefficients would imply – could be removed
Fig 4A-G and Fig 5A-G could be combined into one "comparative microscopy" picture (and 4H-K and 5H-L combined into a single *frame* as suggested above).
Fig 6 demonstrates the validity of the method – important, but not central to the main-text narrative – could go to SI?
Fig 9B-C and Fig 10A,C could be combined into a single panel, then Figs 9 and 10 could potentially be merged.

A connection should be made with
https://journals.biologists.com/dev/article/144/23/4386/19257/Pavement-cells-and-the-topology-puzzle
which, while not focusing specifically on giant cells, does consider generative mechanisms and development of size distributions in pavement cells and their connectivity statistics.
https://www.cambridge.org/core/journals/quantitative-plant-biology/article/using-quantitative-methods-to-understand-leaf-epidermal-development/26AFBE802A863313E7EDD814A477691B
also provides a good overview of existing quantitative approaches to the question.

Scientific comments

L154 – what functional form do the tails of these distributions have? Exponential decay? If they are instead heavier (e.g. power-law) it could speak to an interesting mechanism of generation

L161 – H2B-TFP fluorescence is used as a proxy for nuclear DNA ploidy. Why does it have such a continuous distribution (horizontal axis of Fig 2G-H)? Wouldn't we expect to see somewhat discrete jumps corresponding to integer / 2^n values? If it's because of noise in the readout, does this challenge the interpretation?

L187 and throughout – as above, please plot all cell areas on a logarithmic scale – they invariably spans many orders of magnitude and we lose a lot of info from their "clumping" at low values!

L187 (here and generally) – area is used synonymously with size. So is area a good readout of volume? Does it matter, in terms of generating/control mechanisms? It's fine if this is all a theory of cell area as opposed to size (which usually means volume) – but I couldn't find this mentioned explicitly.

L201 – "the large cells on the blade on one side frequently did not form directly opposite the large cells on the blade on the other side". If this is to be a scientific result, it needs quantification with respect to a reasonable null hypothesis. From eyeballing Fig 3C it actually looks to me like there is some notable symmetry in the large cell spatial distributions – especially given the noise in the overall cell layout. The null hypothesis "every large cell must be exactly opposite another one" is too precise to be interesting; what values of which test statistic would lead the authors to accept that there was

symmetry? (NB, scientifically I believe the large cells are established indepedently – what mechanism could cross the leaf? – but statistically it's not safe to use this as evidence in its current form)

L260 – This MDS plotting (methods l730-733). What is the deal with the normalisation? Every "replicate" is scaled to have average (mean?) 1 – is that every instance of every mutant line in every tissue type? What happens if you don't do this? You're losing a tremendous amount of information about cell size, which clearly varies a lot across cases. If the unnormalised version doesn't show the same clustering structure, then the genetic dependence of shape is rather higher-order than the genetic dependence of size – which should be mentioned if so!

L340 – **The randomisation method (point 1).** This is probably my biggest concern with the paper from here on. For the pavement cells, the randomisation produces cell geometries that look very much more convex that the biology (see pic below). This is clear from the examples given across all the pavement cell sets in Figs 7 and 8. This will certainly affect the statistics of connectivity between cells, as sets of cells with less convoluted boundaries will have fewer opportunities to contact each other. For the sepal cells, the randomisation destroys linear structure in the tissue (seen in the computational example in Fig. 9C). This might also influence the connectivity statistics for giant cells, as it likely gives more opportunities for small cells to sneak in between them than is actually the case in the adjacent ranks seen in the real bio.

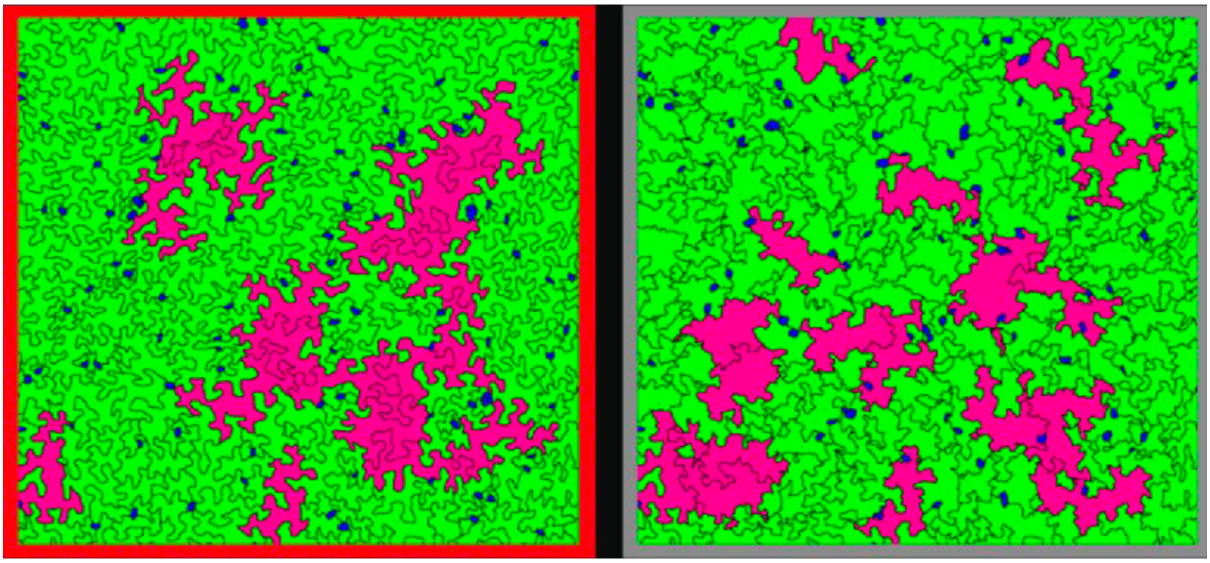

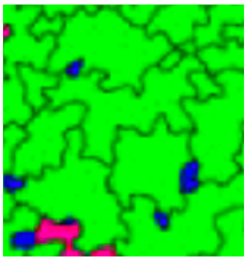

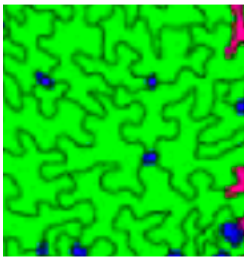

Because of these rather dramatic differences in physical arrangement, I think the randomisation method risks introducing fairly substantial artefacts into the biological structure of the null models. My concern is that the effects reported as departures from the random null model are in the direction of what we'd expect from such artefacts – and as such it's hard to say whether we're seeing signals of a true biological effect, or a comparison with an imperfect null model.

As a reviewer I can't drop a fairly strong criticism like this without proposing a possible solution. It is clearly impossible to pick up a bunch of jigsaw pieces, rearrange them, and put them back into a new and perfect tesselation that fills the original plane. But I wonder if a randomisation method that removes shared sections and introduces new division planes would come close. For example, in the image below, remove boundary between red points and add boundary between yellow points:

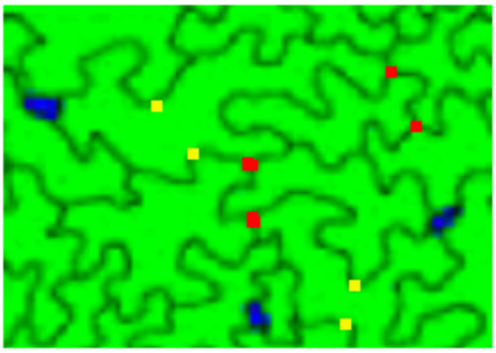

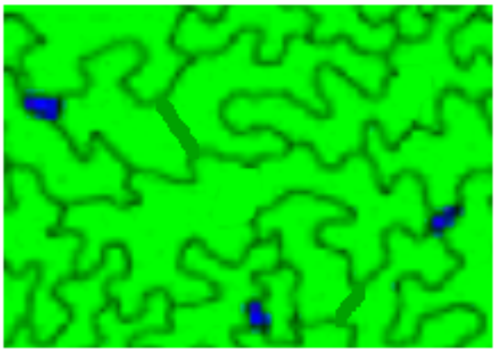

This way you're destroying some giant cells and creating others in arbitrary positions through the cell while preserving the key morphology of pavement cells. This would also

of course work for the sepal cells, redistributing the giant cells while preserving the side-by-side linear rows. At the very least, if this gave similar results to the existing randomisation approach, it could be claimed that two different choices for randomisation agreed in their outputs.

L354 – "In addition, it
was less probable to find isolated giant cells, and more probable to find giant cells in contact with
two or more other giant cells compared with what was expected by chance (Fig. 7D)." – not really in addition, as once we know this statement we know that the mean must be higher.

Fig. 7C, 8B, 8H – we can't have p = 0 here, only p < 0.0025.

L385 – "regulated by a self-catalytic feedback loop" – but this isn't shown in the figure (9A)?

L387 – some details of how cell contents are inherited at divisions, and how spatial structure emerges in the model (do cells push each other out of the way?) is essential for interpretation here (see comments on Methods).

L414 – first – what is the difference between Fig 9D and Fig 10B (right)? Don't they both show giant neighbour counts in simulated tissues at the final time point? Why are they different?
Second – so, if I understand, the argument isn't that giant cells acquire more neighbours over time (indeed Fig. 10A shows the giant interactions are preserved and the red bars in Figs. 10B and D look like they have the same values) – but that the randomised null model gives progressively lower giant cell neighbour counts as time progresses.
But doesn't this mean **(randomisation method point 2)**:

- that giant cells *are* positioned randomly in space – because that's how they simulations start
- that they *remain* positioned randomly in space during development – because the statistics of their neighbouring interactions with other giant cells (irrespective of any null model) don't change
- that randomly swapping cell identities becomes a worse null model as the tissue develops, leading to giant cells departing more from its predictions? This is what we'd expect, because the developmental history of a cell in a tissue influences its physical situation, and so the extent to which a cell is not just a random independent object grows with its history.

I think a fundamental point here is that "agreement with a null model that randomises cells" is taken as synonymous with "randomly arranged", and "departure from a null model that randomises cells" is taken as synonymous with "clustered". But actually the degree of clustering – the connectivity of the giant cells – doesn't change, and nor does their arrangement within the tissue (as seen in simulation in Fig 10A and in real bio in Fig 10C). So it can't be meaningful to say "more clustered" over time (l403, 407, 426, 455) – the change is in the null model predictions, not the direct observations.

It feels like a "quenching" picture, to borrow from physics – interactions between giant cells are quenched while the entropy of the rest of the tissue increases through the constant churn of cell divisions. They're not getting more clustered, but the order they remember from the simpler conditions gets more and more different with respect to the increasing entropy of the tissue as a whole. This is still an interesting message, but I suggest the phrasing be changed throughout to reflect the static nature of the giant cell statistics and the dynamic nature of everything else.

Methods

I couldn't find a link to the software implementation.

For NetworkX, matplotlib, seaborn, and any other libraries involved, please cite the appropriate references to credit the authors in a trackable way rather than just giving a URL.

I think many people who otherwise find the paper interesting will be put off by how the math model is presented on l876-894. It assumes a lot of familiarity with how (stochastic) systems biology models are constructed. Why not have a sentence or two describing what these complicated-looking expressions are actually doing? E.g. "the terms on the right hand side of Eqn 1 describe constitutive expression, self-activation (using an enzyme-kinetic model), degradation, and a (scaled) random term modelling thermodynamic fluctuations". NB – the inline expression on l893 is illegible in my review copy but I'm assuming it's describing zero-autocorrelation unit noise.

Where do the parameter values come from, and how do we know they're reasonable?

What are the inheritance rules for *ATML1* and *Target*? i.e. when a cell divides, are the levels of these variables reset, inherited proportional to daughter cell volume, inherited equally, …? This would seem to be essential in considering spatial correlations in cell behaviour across a growing tissue.

How does spatial structure emerge? Looking at Fig 9B I can see some cells that have divided with a vertical (in the figure) division plane. What's to stop this occuring more than once and pushing apart the lateral structure of the tissue? Why do the giant cells (and others) have thickness differences (tapering)? It seems like there's a tremendous amount of detail here that is swept into "the multicellular tissue grows anisotropically" (L910).
