# Supplementary material for: A common pathway controls cell size in the sepal and leaf epidermis leading to a nonrandom pattern of giant cells
Source: PLoS Biol. 2025 Nov 3;23(11):e3003469. doi: 10.1371/journal.pbio.3003469 (PMC12599956; doi:10.1371/journal.pbio.3003469)
Supplement: Attachment — Submitted filename: Response to reviewers.pdf [file pbio.3003469.s026.pdf]

Editor comments:
The reviewers are attached below. As you will see, the reviewers find the paper beautifully done and very informative for researchers working in the field, however they also raise several issues. Reviewer 1 is not convinced of the overall advance over previous publications, but also thinks that the description of the cell size and shape throughout development on both sides of the leaf is interesting for the field. Reviewer 2 raises issues with the presentation, as the paper is challenging to read, and makes several suggestions to improve it and avoid repetition of the data and better comparisons. This reviewer also mentions that the randomisation method used is not clear and is not convinced that it works for pavement cells as it can introduce artifacts into the biological structure of the null models, and suggests a different method that removes shared sections and introduces new division planes. In addition, the reviewer thinks that instead of the cells getting more clustered, as proposed, the order they remember from the simpler conditions could get more and more different with respect to the increasing entropy of the tissue as a whole. You should consider this interpretation of the results.

Thank you. We have addressed all of these points as described in detail below in the response to reviewers.

Rev. 1:

In this paper, the authors analyse the distribution of giant cells in arabidopsis leaves and show that the same genetic pathway controls giant cell formation in both leaves and sepals. They also generate a computational model that explains how initially randomly distributed giant cell precursors transition to a clustered, non-random distribution.

The work is carefully done and clearly explained, with beautiful images of leaves beautifully quantified. The authors have also gone to considerable effort to quantify development on the upper and lower surfaces of the leaf. This is rarely done, and gives a much fuller view of leaf patterning. As a description of epidermal cell patterning in leaf development, it is outstanding. However, the scientific advance (that the same genetic pathway regulates giant cells in the leaf as well as the sepal) seems fairly minor.

It is already known that lgo mutants have altered pavement cell division, size and endoreduplication in leaves and that LGO's paralogues in the SIM/SMR family alter pavement cell size (Roeder et al. 2010, Churchman et al. 2006, Kumar et al. 2015 and Dubois et al. 2023). The model presented here is essentially the same as one already published by the authors and is known to explain the distribution of giant cells in sepals (Meyer et al. 2017). Given this previous data, the finding that the same pathway works to regulate giant cells in leaves as well as sepals seems unsurprising. The beautiful description of cell size and shape throughout development on both sides of the leaf will be of interest to other researchers in the field though.

We agree that the dataset and its quantitative description presented in this manuscript will be of interest to other researchers in the field.

Previously, it was known that there are endoreplicated cells in the leaf epidermis, and that *LGO* is involved in their endoreduplication, as the reviewer states; however, the finding that the rest of the genes in this giant cell specification genetic pathway also control the developmental formation of endoreduplicated cells in the leaf was not known. We agree that the genetic pathway is the same in leaves and sepals, which we think is also interesting. Previously, in the study of leaves, these cells have been presented as a cell cycle/endoreduplication pathway as opposed to a developmental pathway, so our demonstration that these cells are formed through a developmental pathway is novel as well. Likewise, the definition of these highly endoreduplicated cells as giant cells, a specialized cell type, is also an important point of this paper.

Furthermore, our analysis of the randomness of the spatial arrangement of giant cells is novel.

Here, we are applying the model from Meyer et al., 2017 to determine whether it can recapitulate the cell size spatial patterning. Previously, the model was only shown to reproduce dynamical aspects of the ATML1 fluctuations and the proportion of different cell ploidies, not the spatial patterning. Yes, this is not a new model, but that makes our conclusion more powerful because our existing model with no further optimization, already reproduces the random to nonrandom shift in the spatial pattern of giant cells over developmental time.

Rev. 2: Iain Johnston – note that this reviewer has signed his review.

This is a detailed survey of an interesting and deep aspect of plant biology – the control of physical properties of cell size and positioning. Some beautiful microscopy and comprehensive mutant analysis paints a picture connecting the same circuitry to cell size control in two different cases. Computational and modelling theory is developed to explore evidence for cell properties departing from random null models, and to explore a putative explanatory mechanism for the links observed.

I enjoyed reading this paper and think its survey of physical properties and their genetic controllers is an interesting story. I have some comments about the presentation and some concerns about the randomization-based null models (and hence the results downstream of these), but these may well be due to my own misunderstanding and I'd be very open to being corrected. The most important scientific comments are in bold as "**randomisation method point 1 & 2"** below.

For transparency, I am Iain Johnston and I am happy for this review to be treated as public domain. To my eyes my most important shortcomings as a reviewer are a lack of familiarity with the genetic players involved in the regulatory circuit under study.

Thanks for the positive comments. We address the points raised below.

Presentation comments

This is a huge paper and as such it is a challenge to read. That's fine – readers should be challenged. But the challenge is compounded by the placement and styling of important information. With this many figures, the reader could be helped substantially more.

We are many times directed to multiple subfigures of different figures to support a scientific point (e.g. "the size of the largest cells is moderately reduced in *acr4-2* mutants (Figs. 1B, 4B, 4H–J, 5B, 5H–I, 216 K), and more greatly reduced in *atml1-3* mutants (Figs. 1C, 4C, 4H–J, 5C, 5H–I, K).", l215-6; also l222, 250, 255, 269, 272, 278, 298, perhaps some more). I'm sure I'm not alone in finding it challenging to make comparisons across multiple subfigures across multiple pages.

First – parts 4HIJ and 5HIK are the same data many times over and the best way of plotting these data is actually none of these options – they should be visualised as individual datapoints, with their profile expanded to reflect density (e.g. with ggbeeswarm), with the cell area scale on a logarithmic axis. This is also the case for 3D, 2E, 2F, and any other distributions of cell area in the SI.

Second – to my eyes the most important message from these comparisons is the similarities (or differences) in the impact of the different mutants across the tissue types (sepal, early leaves, mature leaves). This could be done in one frame. Fig 5L is the best current example, but I'm confused about the normalisation (see below). Why not have pointwise, log-scaled, cell area distributions in a trellis plot for mutants (columns) and tissues (row)? Then the reader can scan across the trellis, see the similar patterns across tissues from the different genetic perturbations, and get all the information together.

Thank you for these suggestions. We have now combined Figs. 4 and 5 into a new figure (now Fig. 4) for a direct comparison between 9-dpg and 25 dpg-leaves. We have also changed the panel labels for easier reference. To facilitate visualization, we now plot the cell size distributions of the three replicates together in Fig. 4C-D. The individual replicate distributions are shown in Fig. S8A-B.

In the previous version of the manuscript, the linear scale was chosen to emphasize the tail of the distributions which contain the giant cells. We propose to keep a linear scale in Fig. 2E to analyze and compare the tails of leaf and sepal cell sizes. However, in the new Figs. 3 and 4 (previous Figs. 3, 4 and 5), where the entire distributions are analyzed, the cell size distributions are now plotted on a log scale. As the large number of points made it difficult to visualize them all with a swarm plot, we opted for violin plots instead. See the alternative swarm plot versions below for 25-dpg leaves:

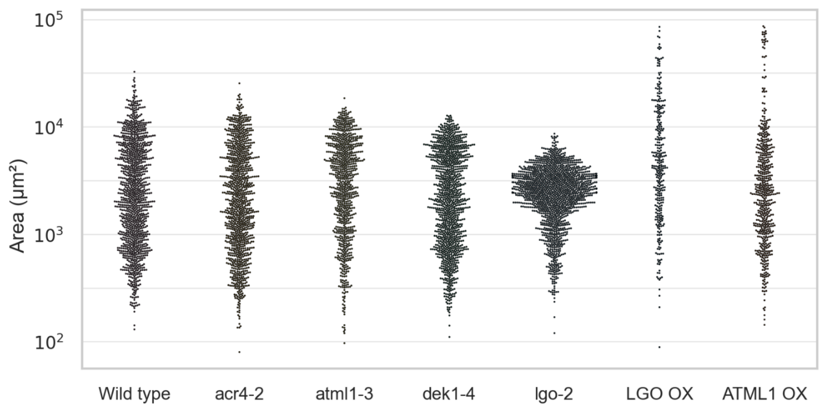

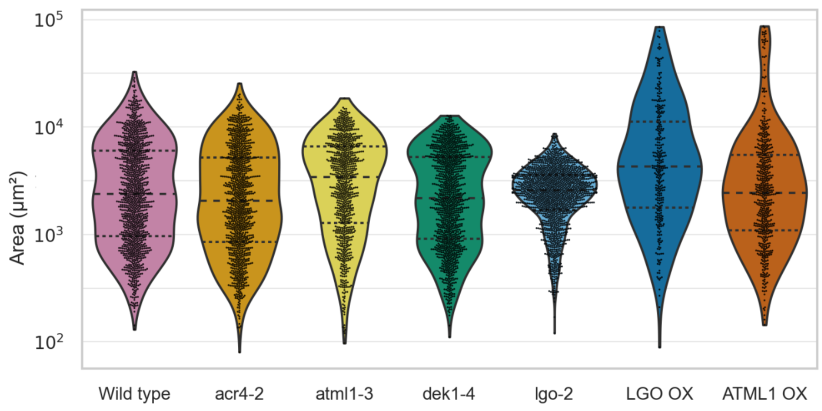

Some suggestions for paring back content:
Fig 2GH don't report much (anything?) more than their correlation coefficients would imply – could be removed

Thanks - We have updated Fig. 2 and improved the plots. We still believe that it is important to show the differences between the adaxial and abaxial sides (the former Fig. 2G is now Fig. 2F). However, we have moved the former Fig. 2H to supplementary Fig. S2H (and the previous panel from Fig. S2H is now merged with Fig. S2G).

Please note that we have also updated Fig. 3. Cell area distributions are now shown on a log scale with a violin plot. We also only show the tails of the distributions (largest cells). Below, if needed, you can find the whole cell size distributions (the y axis is the exponent to 10):

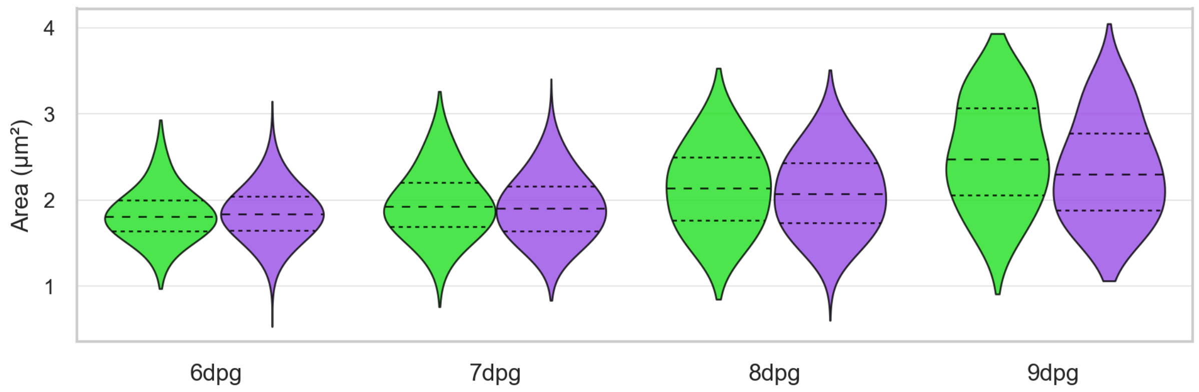

Fig 4A-G and Fig 5A-G could be combined into one “comparative microscopy” picture (and 4H-K and 5H-L combined into a single *frame* as suggested above).

Many thanks - we have merged Figs. 4 and 5, which are now the new Fig. 4, as explained above.

Fig 6 demonstrates the validity of the method – important, but not central to the main text narrative – could go to SI?

Thanks. We have moved the previous Fig. 6 to the supplement as the new Fig. S10.

Fig 9B-C and Fig 10A,C could be combined into a single panel, then Figs 9 and 10 could potentially be merged.

Many thanks - we have merged Figs. 9 and 10, they are now the new Fig. 7.

A connection should be made with
https://journals.biologists.com/dev/article/144/23/4386/19257/Pavement-cells-and the-topology-puzzle
which, while not focusing specifically on giant cells, does consider generative mechanisms and development of size distributions in pavement cells and their connectivity statistics.
https://www.cambridge.org/core/journals/quantitative-plant-biology/article/using quantitative-methods-to-understand-leaf-epidermal development/26AFBE802A863313E7EDD814A477691B
also provides a good overview of existing quantitative approaches to the question.

The first mentioned article, Carter et al. 2017, is relevant because the authors study the relation between topology and cell size. However, it focuses on *spch* mutants, which lack stomata and their small stomatal lineage cells, and on young leaves, where giant cells are not observed. In this article, the authors study for example the relation between cell areas and cell areas of neighboring cells and found a correlation: larger cells tend to be surrounded by smaller ones, as expected from entropic considerations. This observation contrasts with our results in wild type (Fig. 6), where large cells tend

to cluster, medium-sized cells show no deviation from randomness, and small pavement cells also cluster. To explore this, we produced an additional plot (now Fig. S18D) analyzing the relationship between cell size and the average size of neighboring cells across the six experimental replicates (with just one randomization per replicate). We refer to this in the manuscript, where we also cite Carter et al. 2017:

“It was previously shown that, apart from small cells, pavement cells follow the theoretical topological laws based on space-filling (i.e., via entropic considerations), with larger cells being on average surrounded by smaller ones in young *spch* leaf tissues (37). Our analyses on more mature wild-type leaves reveal that larger cells are surrounded by larger cells (and have fewer neighbors) than what is expected by purely random space-filling given by our null model (Fig. S18D, Fig. 6). This highlights the existence of a regulated biological mechanism governing cell size and their spatial arrangement.“

We have also cited the second suggested review, Kuan et al. (2022).

Scientific comments

L154 – what functional form do the tails of these distributions have? Exponential decay? If they are instead heavier (e.g. power-law) it could speak to an interesting mechanism of generation

This is an interesting point; we found that all genotypes showed an exponential decay (i.e., the cumulative distribution of the area was following a log-linear relation) except the ATML1 OX, which presumably has a heavier tail. However, *lgo-2*, *atml1-3*, and *dek1-4* show a more abrupt drop to zero in the log-linear plot compared to wild type and *acr4-2*, likely due to a reduced number of giant cell outliers. From the log-log plot, we do not see any clear power law. Although we find these are interesting behaviours, we feel the paper is already quite dense for a general audience, and we prefer to leave this quantification out for the sake of focusing on our main findings. See plots below.

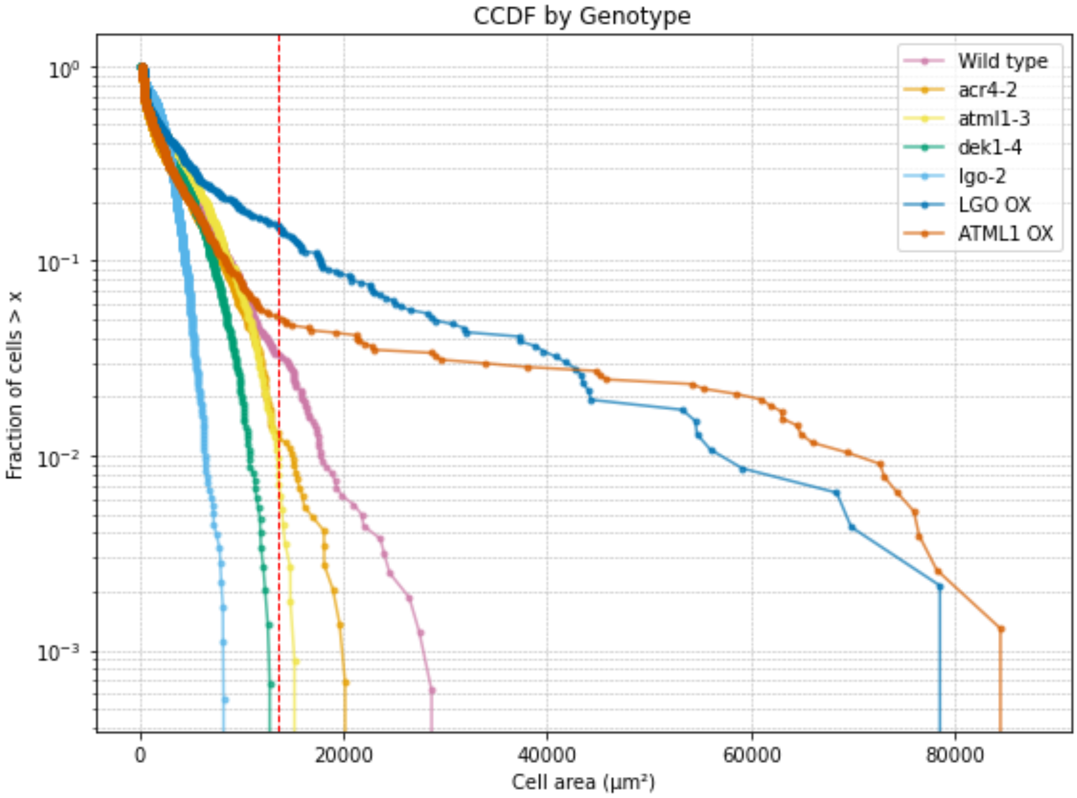
CCDF by Genotype
Wild type
acr4-2
atml1-3
dek1-4
lgo-2
LGO OX
ATML1 OX
Fraction of cells > x
Cell area (μm²)
0
20000
40000
60000
80000

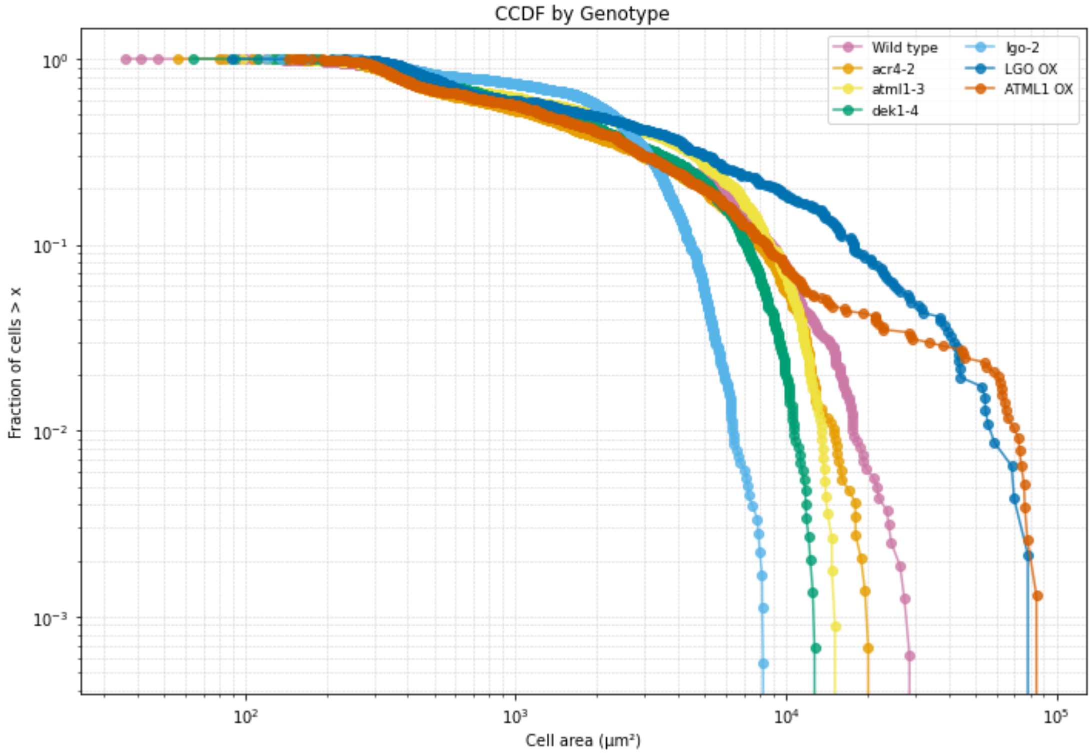
CCDF by Genotype
Wild type
acr4-2
atml1-3
dek1-4
lgo-2
LGO OX
ATML1 OX
Fraction of cells > x
Cell area (μm²)

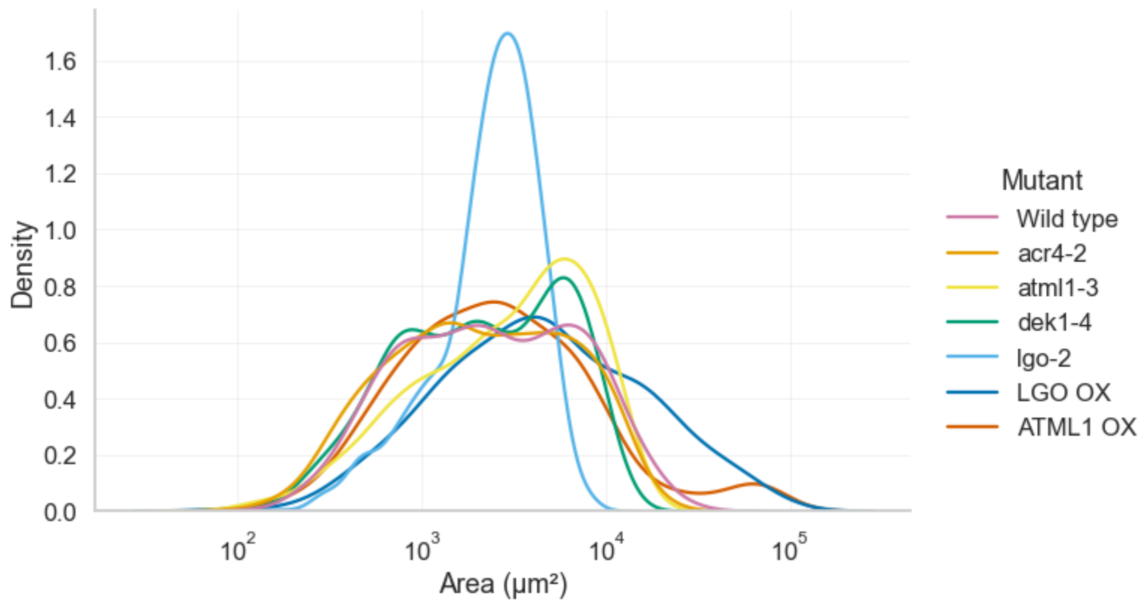

L161 – H2B-TFP fluorescence is used as a proxy for nuclear DNA ploidy. Why does it have such a continuous distribution (horizontal axis of Fig 2G-H)? Wouldn't we expect to see somewhat discrete jumps corresponding to integer / 2^n values? If it's because of noise in the readout, does this challenge the interpretation?

This is a good point. In a new manuscript (Russell et al. 2025 bioRxiv, https://doi.org/10.1101/2025.03.11.642217), we indeed find that, if there are enough data points, the distribution of histone nuclear concentrations shows distinct peaks, which can be eventually related to ploidy. We now refer to the other manuscript for clarifying this point.

"A recent study showed that fluorescence levels of histone fluorescence reporters measured from microscopy images are a good proxy to infer nuclear ploidy in Arabidopsis cotyledons (28). Hence, to validate whether cell area and ploidy were correlated in our leaves, we measured DNA content by quantifying total fluorescence of Histone 2B-TFP (*pUBQ10::H2B-TFP*) within each cell nucleus (...)"

L187 and throughout – as above, please plot all cell areas on a logarithmic scale – they invariably spans many orders of magnitude and we lose a lot of info from their "clumping" at low values!

We have now plotted all cell area distributions on a log scale (apart from Fig. 2, where we wanted to focus on the tails of the distributions).

L187 (here and generally) – area is used synonymously with size. So is area a good readout of volume? Does it matter, in terms of generating/control mechanisms? It's fine if this is all a theory of cell area as opposed to size (which usually means volume) – but I couldn't find this mentioned explicitly.

We and others would argue that area is a more relevant 'size measure' than volume in plant epidermal cells (Melaragno et al., 1993; Robinson et al., 2018). Melaragno et

al found that cell surface area scaled more closely with ploidy than volume. Plant epidermal cells are almost entirely filled by a large central vacuole, so the cytoplasm forms a thin layer around the edge of the cell. Thus, the volume of the cytoplasm is better approximated by the surface area of the cell. It is cytoplasmic volume, not cell volume that is thought to scale with endopolyploidy, making cell surface area, and by proxy cell area a good measure of size. Therefore, we have clarified in the text that we use size in our case to refer to area:

"Throughout our analysis, we use the term size to mean cell area because area has been shown to be a more relevant measure of cell size than volume in highly vacuolated plant epidermal cells (Melaragno et al., 1993).".

L201 – "the large cells on the blade on one side frequently did not form directly opposite the large cells on the blade on the other side". If this is to be a scientific result, it needs quantification with respect to a reasonable null hypothesis. From eyeballing Fig 3C it actually looks to me like there is some notable symmetry in the large cell spatial distributions – especially given the noise in the overall cell layout. The null hypothesis "every large cell must be exactly opposite another one" is too precise to be interesting; what values of which test statistic would lead the authors to accept that there was symmetry? (NB, scientifically I believe the large cells are established independently – what mechanism could cross the leaf? – but statistically it's not safe to use this as evidence in its current form)

In that sentence we did mean giant cells that are directly, exactly across from one another on opposite sides of the leaf, which we do think is an interesting null hypothesis. There are signals that cross the abaxial-adaxial axis of the leaf. For example, microRNA166 forms a gradient from the abaxial epidermis inward toward the adaxial epidermis, whereas tasiARF small RNAs form an opposing gradient from the adaxial side toward the abaxial side (Skopelitis et al., 2017, doi:10.1016/j.devcel.2017.10.003). Likewise, if there were some internal tissue signal such as auxin or some other signal emanating from the vasculature, we would expect to see giant cells across from each other in the leaf, directly overlying the vasculature, which we test later. However, we agree it is difficult to quantify the exact spatial relationship between cells on each side of the leaf, so we have removed this statement.

We do agree there is an overarching pattern on both sides of the leaf, which is that giant cells are formed in the basipetal differentiation gradient, and therefore giant cells on both sides of the leaf form above the same lower border on both sides of the leaf. We have left this point in the manuscript.

L260 – This MDS plotting (methods l730-733). What is the deal with the normalisation? Every "replicate" is scaled to have average (mean?) 1 – is that every instance of every mutant line in every tissue type? What happens if you don't do this? You're losing a tremendous amount of information about cell size, which clearly varies a lot across

cases. If the unnormalised version doesn't show the same clustering structure, then the genetic dependence of shape is rather higher-order than the genetic dependence of size – which should be mentioned if so!

Note we do show MDS plots at single days which are not normalised to 1, and have been moved to the supplement as Figures S4H and S6H.

Regarding the MDS normalized plot (now Figure 4E), we have normalized it to be able to compare across the 9 day and 25-day samples. The normalization is indeed for every replicate in every genotype, as explained in the text. The cell sizes change substantially between these days, so normalization is essential to be able to compare the samples across those days. This is explained in the statistical analysis section of the methods:
"To create the Wasserstein plot of the combined 9-dpg and 25-dpg cell area data, cell areas of each replicate in each genotype were normalized by the mean cell area for that replicate. In this way, each replicate has a mean of 1. This eliminated the difference in the values of the cell size between the 9-dpg and 25-dpg leaves, such that the variances of the cell areas can be compared rather than the absolute sizes."

The same trends are seen in the normalized and the non-normalized plots.

L340 – **The randomisation method (point 1).** This is probably my biggest concern with the paper from here on. For the pavement cells, the randomisation produces cell geometries that look very much more convex that the biology (see pic below). This is clear from the examples given across all the pavement cell sets in Figs 7 and 8. This will certainly affect the statistics of connectivity between cells, as sets of cells with less convoluted boundaries will have fewer opportunities to contact each other. For the sepal cells, the randomisation destroys linear structure in the tissue (seen in the computational example in Fig. 9C). This might also influence the connectivity statistics for giant cells, as it likely gives more opportunities for small cells to sneak in between them than is actually the case in the adjacent ranks seen in the real bio.

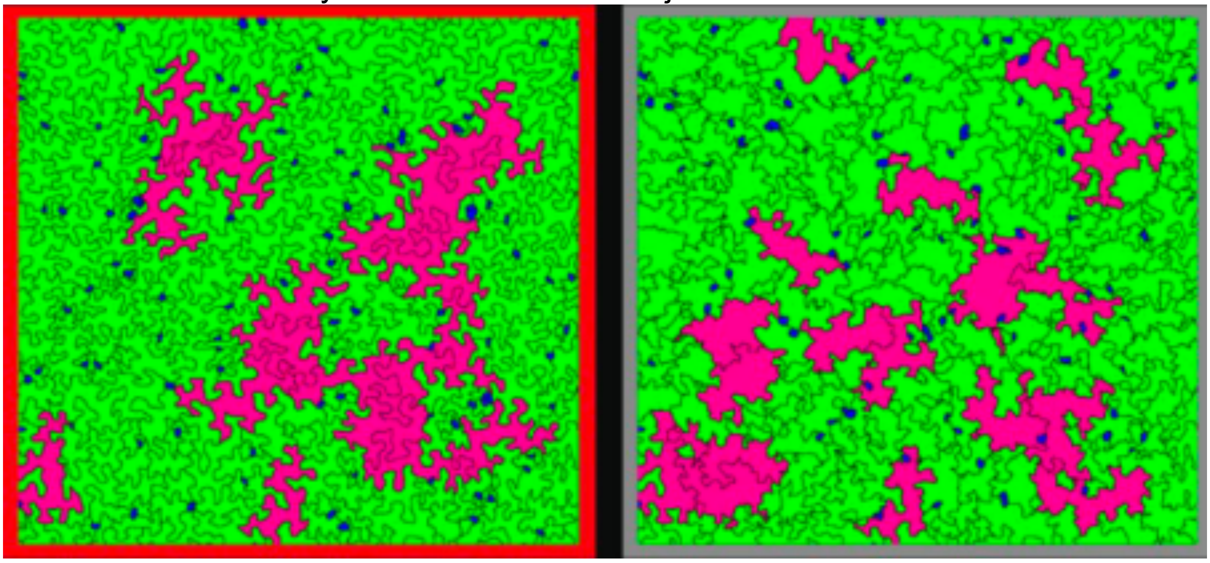

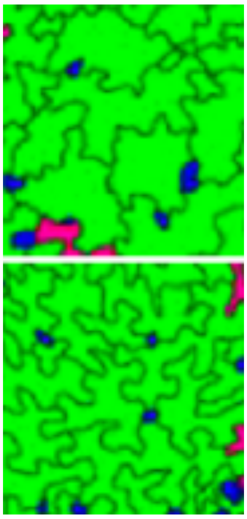

Because of these rather dramatic differences in physical arrangement, I think the randomisation method risks introducing fairly substantial artefacts into the biological structure of the null models. My concern is that the effects reported as departures from the random null model are in the direction of what we'd expect from such artefacts – and as such it's hard to say whether we're seeing signals of a true biological effect, or a comparison with an imperfect null model.

It is a fair point that could potentially introduce bias in our null model. We acknowledge now more clearly that cell shape is affected in our randomized tissues, specially in the leaf (Fig. S15): "In the randomized tissues, cell sizes were well preserved (Fig. S15D), but cell shapes were slightly affected in the leaf (Fig. S15E)". However, we think that these artifacts do not significantly affect our results.

To evaluate the potential bias in the null model, we previously tested it on a randomly selected population of pavement cells as well as other cell size populations (Fig. 6) in the leaf and found no major bias: the quantified observable from the segmentations shown by the red bar fell near the center of the null distribution for randomly selected cells. We have now extended this test on different populations (5 distinct populations) of randomly selected pavement cells in both leaves and sepals (Fig. S17). If pavement cells would make less contacts on average in the randomized tissues due to their more convex shapes, we would expect the red bar to be shifted to the right side of the null distribution. No major bias was found in the leaf (Fig. S17A-B) nor in the sepal (Fig. S17C-D) randomized tissues, as we did not reject the null hypothesis on these artificial random patterns. See lines 1013-1020

To investigate in detail whether the introduced cell shape artifacts impact the cell connectivity and the results in leaves, we also reconstructed the original tissues with similar shape artefacts to those in the randomized tissues (Fig. S18). Note that the generation of a "reconstruction" was also performed in the originally published SET article (Laruelle et al, Commun Biol 2020) to enable a direct comparison with the randomizations. These new tissues (called reconstructions) were generated using the same image-based dmSET method (Fig. S18A-B), but by constraining the cell positions to their initial positions with some added noise. The introduced shape artifacts did not significantly alter the connectivity of the large cells. When comparing the reconstructions with the randomizations, our conclusions about giant cells being

more clustered than what would be expected by chance remain the same (Fig. S18O). See more details in Materials and Methods lines 992-1011: "To explore this possibility, we generated "reconstructions" of the original images using the same dmSET method as for the randomizations, except that each cell's position from the initial segmentation was preserved (with some added noise) (Fig. S18A–C, E–F). This resulted in cell shape artifacts that were nearly identical between the reconstructions and the randomizations (Fig. S18J–L), making them more comparable. Indeed, the differences in cell lobeyness (defined as the perimeter of the cell divided by the perimeter of its convex hull) from the original tissues were similar between the reconstructions and randomizations for large cells(Fig. S18J). The number of cell neighbors differed slightly from the original segmentations due to the introduced shape artifacts (Fig. S18M–N), but we found that giant cell contacts remained mostly the same in the reconstructions. Specifically, we observed approximately 6% fewer contacts between giant cells in the reconstructions compared with the segmentations. When comparing reconstructions with randomizations (which are comparable because of their similar cell shapes), the results remained consistent (Fig. S18O), with giant cells being more clustered than expected by chance (p=0.005)."

As a reviewer I can't drop a fairly strong criticism like this without proposing a possible solution. It is clearly impossible to pick up a bunch of jigsaw pieces, rearrange them, and put them back into a new and perfect tesselation that fills the original plane. But I wonder if a randomisation method that removes shared sections and introduces new division planes would come close. For example, in the image below, remove boundary between red points and add boundary between yellow points:

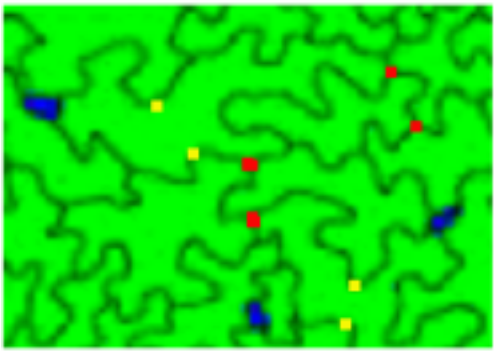

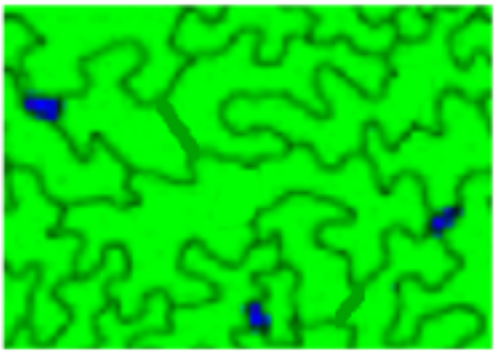

This way you're destroying some giant cells and creating others in arbitrary positions through the cell while preserving the key morphology of pavement cells. This would also of course work for the sepal cells, redistributing the giant cells while preserving the side-by-side linear rows. At the very least, if this gave similar results to the existing

randomisation approach, it could be claimed that two different choices for randomisation agreed in their outputs.

We highly appreciate the suggestion of the alternative method, and we also think this needs to be performed such that the cell size distribution is unperturbed in the tissue, as done with the dmSET method.

We implemented this new method, which we called CMC (Cut and Merge Cells) and applied it to the leaf epidermis, which presents the most concerning shape artifacts (Fig. S19). The results are shown in Fig. S19B-D. Details on the implementation are provided in the Materials and Methods (see below) section and in the figure below: "Because the leaf pavement cells in the randomizations exhibit different shapes (more convex shapes and noisier edges) compared to the original cells, we developed an additional alternative method for generating randomized tissues (Fig. S19), called CMC (Cut and Merge Cells). In this approach, each leaf replicate was first over-segmented using MorphoGraphX to create templates composed of small fragments of pavement cells (Fig. S19A). These templates were then used to automatically generate a random pattern of giant cells, preserving their original sizes and numbers (Fig. 19A). Giant cells were created recursively: their positions were randomly allocated to a first pavement cell fragment, which was then merged with neighboring cell fragments until the original giant cell size was reached (Fig. 19A). Similar results were obtained by using this randomization method (Fig. 19B–C), confirming the tendency of giant cells to cluster more than would be expected by chance."

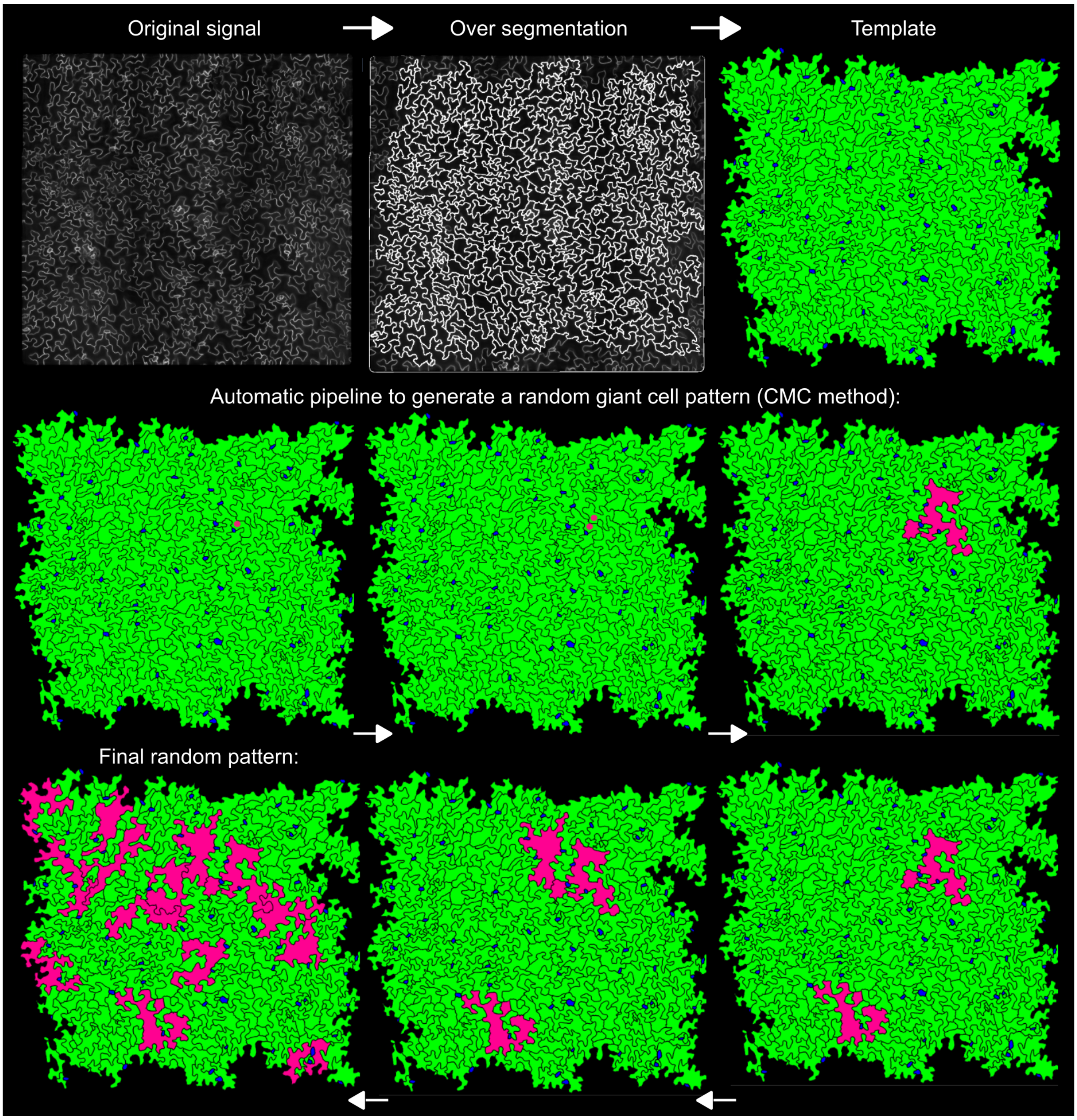

Using this alternative CMC method on all leaf replicates, we found very similar results (Fig S19B-D), which supports the idea that giant cells are more clustered than would be expected by chance. However, the shape of the created giant cells is not perfectly preserved either with this method (Fig S19F), even if the artificially created giant cells “looks more” like pavement cells. We think that accurately reproducing the exact shape of each cell, as intended with the dmSET method, would be very challenging and could likely introduce other types of shape artifacts. Moreover, this CMC approach does not allow for shuffling all cells, including stomata. Yet, it is reassuring that this additional method, although not being perfect, would lead to the same conclusions.

Therefore, we decided to present the CMC method as a supplementary figure to demonstrate that an independent approach produces comparable results. In the current version of the article, we now acknowledge the shape artifacts introduced by the dmSET method, and show that these artifacts (see lines 992-1020):

1) Do not significantly impact the connectivity between giant cells in leaves. By generating reconstructed tissues where cell shapes are affected in a similar manner than in the randomized tissues, we found no significant difference in giant cell connectivity (Fig. S18M-N) and in our results (Fig. S18O). See our previous answer and lines.
2) Do not introduce a significant bias in the null models, as the controls performed on randomly selected pavement cell populations failed to reject the null hypothesis.See our previous answer and lines.

We also refer to this in the results section: "To ensure that these shape artifacts did not introduce bias in our analyses, we tested our method on a randomly selected population of cells in both leaves and sepals (see Materials and Methods) and confirmed the absence of significant bias in the null models (Fig. S17). Additionally, we reconstructed the original leaf tissues with shape artifacts similar to those in the randomized tissues (Fig. S18), and found that giant cell connectivity was largely preserved and that the results remained consistent (Fig. S18) (see Materials and Methods)."

In the sepal, we think that the structure and cell shape are reasonably well preserved, which we support with some additional panels (Fig. S15F-J), and that potential shape artifacts in the randomized tissues would potentially only increase the number of giant cell contacts, thus not changing our conclusions. Indeed, cells have slightly noisier edges with a higher perimeter (Fig. S15J) and/or may be tilted due to the preservation of the original orientation. Both effects would increase the contact probability - and again, these effects would not challenge our conclusions, but the opposite. Although the linear structure is not perfectly preserved, we note that real sepals also contain irregular and misaligned cells. In addition, the preservation is better than with the CMC method, which was challenging and produced several additional kinds of cell shape artifacts. Therefore, we are not including it in the manuscript. See below a trial of the CMC method in the sepal:

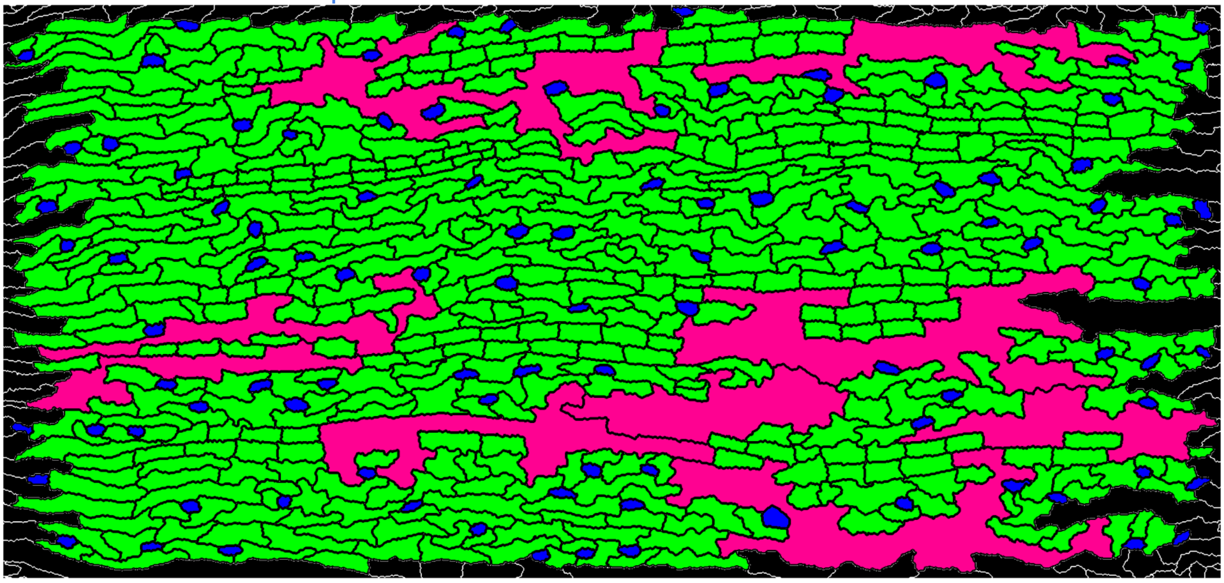

L354 – "In addition, it was less probable to find isolated giant cells, and more probable

to find giant cells in contact with two or more other giant cells compared with what was expected by chance (Fig. 7D)." – not really in addition, as once we know this statement we know that the mean must be higher.

Thanks. We removed "In addition".

Fig. 7C, 8B, 8H – we can't have p = 0 here, only p < 0.0025.

Many thanks for raising this point, we have amended the p-values (see Figs. 5-6) and clarified this in the methods section: "If the value fell outside the null distribution, we assigned $p < 0.0025$, with 0.0025 corresponding to the inverse of the number of random images (400) used to create the null distribution.

L385 – "regulated by a self-catalytic feedback loop" – but this isn't shown in the figure (9A)?

Thanks for noticing it; it was a mistake. The self-catalytic feedback loop is now present in the cartoon of the regulatory network (see Fig. 7A).

L387 – some details of how cell contents are inherited at divisions, and how spatial structure emerges in the model (do cells push each other out of the way?) is essential for interpretation here (see comments on Methods).

See below in the Methods comments in relation to how cell contents are inherited at divisions.

Cells do not push each other, we do not model the mechanics of the system, cell growth is imposed; see below in the comments on Methods on how we implemented growth.

L414 – first – what is the difference between Fig 9D and Fig 10B (right)? Don't they both show giant neighbour counts in simulated tissues at the final time point? Why are they different?

While these two results are produced from the same simulations, the data is not analyzed in the same manner:
- In former Fig. 9D (current Fig. 7D), only a crop section was analyzed and giant cells were defined with a size threshold to make these results comparable with experimental results that were produced in the same way (Fig. 5B-D bottom).
- In former Fig. 10B (current Fig. 7F), the goal was different: to track and quantify the same cell lineage over time. For this reason, the entire tissue was analyzed (without cropping) to ensure that the same cells were included at both time points. In addition, only the giant cells that were already present at the initial time point were considered in the analyses at the final time point, so giant cells were not defined with a threshold but by the giant cell commitment model itself as those cells that would have committed for endoreduplicating at a given time (t=55) or had already endoreduplicated, as explained in the text. We slightly extended the explanation in the corresponding Material and methods:

“only the first-arising giant cells (i.e., cells that stopped dividing after time t = 55 of the simulations for being committed to endoreduplicate) were studied”

Second – so, if I understand, the argument isn’t that giant cells acquire more neighbours over time (indeed Fig. 10A shows the giant interactions are preserved and the red bars in Figs. 10B and D look like they have the same values) – but that the randomised null model gives progressively lower giant cell neighbour counts as time progresses. But doesn’t this mean **(randomisation method point 2)**:

- that giant cells *are* positioned randomly in space – because that’s how they simulations start

- that they *remain* positioned randomly in space during development – because the statistics of their neighbouring interactions with other giant cells (irrespective of any null model) don’t change

  The initial simulated giant cells are positioned randomly in space, as a consequence of the stochastic ATML1 model, and this is supported by the top panel of Fig. 7F. Indeed, the contacts between giant cells are also preserved over time. But if we take into account all the cells and how they are connected, i.e., we take into account the whole cellular network space, then our results show that giant cells do not remain randomly organized in the network space, given the divisions of the non-giant cells. See more details below.

- that randomly swapping cell identities becomes a worse null model as the tissue develops, leading to giant cells departing more from its predictions? This is what we’d expect, because the developmental history of a cell in a tissue influences its physical situation, and so the extent to which a cell is not just a random independent object grows with its history.

I think a fundamental point here is that “agreement with a null model that randomises cells” is taken as synonymous with “randomly arranged”, and “departure from a null model that randomises cells” is taken as synonymous with “clustered”. But actually the degree of clustering – the connectivity of the giant cells – doesn’t change, and nor does their arrangement within the tissue (as seen in simulation in Fig 10A and in real bio in Fig 10C). So it can’t be meaningful to say “more clustered” over time (l403, 407, 426, 455) – the change is in the null model predictions, not the direct observations.

Thanks for these interesting thoughts about the system and its interpretation. We agree that we should be more precise in the terms we use.

- Yes, the “degree of clustering between giant cells” (i.e., the number of contacts between giant cells) doesn’t change. However, the departure from randomness in the null model occurs because, as you noted, the null model considers and randomizes all cells in the tissue. Therefore, it is what we refer now as the giant cell arrangement “within the tissue” (in the context of, or relative to the rest of the tissue) that changes. As it might indeed be confusing to say “more clustered”, we propose to say instead

“more clustered regarding the rest of the tissue” or “in the context of the whole tissue”. Please tell us whether you have a preference.

- Understanding that the giant cell became “more clustered in the context of the whole tissue” is still important for two reasons. Firstly, it explains why we found a clustered giant cell pattern in the mature tissues using our method (Fig. 5). Secondly, it illustrates that a pattern that initially emerges randomly can still appear clustered later (because more cells are added as the tissue grows), emphasizing the importance of dynamics and tissue growth in shaping the resulting cellular structure.

It feels like a “quenching” picture, to borrow from physics – interactions between giant cells are quenched while the entropy of the rest of the tissue increases through the constant churn of cell divisions. They’re not getting more clustered, but the order they remember from the simpler conditions gets more and more different with respect to the increasing entropy of the tissue as a whole. This is still an interesting message, but I suggest the phrasing be changed throughout to reflect the static nature of the giant cell statistics and the dynamic nature of everything else.

We agree this might be possible to justify from the entropic point of view. Yet, for simplicity and for the readership, we feel the above rephrasing we proposed should be sufficient. As pointed above, we agree that the first giant cells that are formed in the sepal are not changing position or connections among them. It is the cell divisions around them that alter the pattern of giant cells relative to the whole tissue. We have clarified this point in the abstract, the end of the introduction, and the discussion.

Methods

I couldn’t find a link to the software implementation.

In the data availability, we referred the reader to DOI: 10.17605/OSF.IO/RFCWS. We have improved the initially uploaded OSF material, and now there is a new folder that enables a better recapitulation of the simulated results (simulations_ATML1 folder).

For NetworkX, matplotlib, seaborn, and any other libraries involved, please cite the appropriate references to credit the authors in a trackable way rather than just giving a URL.

We have added the required references for NetworkX, matplotlib, seaborn and Paraview.

I think many people who otherwise find the paper interesting will be put off by how the math model is presented on l876-894. It assumes a lot of familiarity with how (stochastic) systems biology models are constructed. Why not have a sentence or two describing what these complicated-looking expressions are actually doing? E.g. “the terms on the right hand side of Eqn 1 describe constitutive expression, self-activation

(using an enzyme-kinetic model), degradation, and a (scaled) random term modelling thermodynamic fluctuations". NB – the inline expression on l893 is illegible in my review copy but I'm assuming it's describing zero-autocorrelation unit noise.

Thanks, we have added clarity to the explanation of the model, which reads now:

"To model the dynamics of the concentrations for ATML1, LGO and Timer, we use chemical Langevin equations (59), which are differential equations with a corresponding deterministic part, consisting of production, degradation and regulatory terms, followed by a stochastic part modelling thermodynamic fluctuations that contains a square root, whose radicand has the sum of the absolute values of the production, degradation, and regulatory terms. The dynamics of the ATML1, LGO and Timer concentration in cell i, denoted by $[ATML1]_i$, $[Target]_i$ and $[Timer]_i$ respectively, follow the Lanvegin equations given by

(EQS)

Eqn. (1) stands for the rate of change of ATML1 in cell *i*, and its terms on the right-hand side describe constitutive expression, self-activation (implemented via a Hill function) (Kadiyala et al. 2025), linear degradation, and a stochastic term; Eqn. (2), stands for the rate of change of the Target in cell *i*, its terms on the right-hand side describe ATML1-induced expression of the Target (using also a Hill function), linear degradation, and the corresponding stochastic term in the Target; Eqn. (3) stands for the rate of change of the Timer in cell *i*, and its terms on the right-hand side are a constitutive production and a stochastic term. The parameters of the equations are as follows: (...)"

And yes, there was the correlation function written to describe a Gaussian noise, and we are making sure that the pdf conversion this time it does not pose any formatting problems.

Where do the parameter values come from, and how do we know they're reasonable?

This is a phenomenological model proposed in Meyer et al. 2017. Parameter values were very similar to those from Meyer et al. 2017 chosen to represent the wild-type behaviour. Those parameters were set to values that would lead to a spatially scattered pattern of giant cells, as well as some features that could recapitulate the experimental data shown in Meyer et al. 2017 (the CV of ATML1; the Receiver Operator Analysis referred to as ROC curves, etc.), and we checked we could also reproduce those behaviours. We felt that explaining all those details would not be necessary, but we added the sentence "Parameters were chosen such that the wild-type behaviour reported in Meyer et al. (2017) could be recapitulated."

Those parameters were sufficient to recapitulate the spatial trends observed in the experiments in the current manuscript.

What are the inheritance rules for *ATML1* and *Target*? i.e. when a cell divides, are the levels of these variables reset, inherited proportional to daughter cell volume,

inherited equally, …? This would seem to be essential in considering spatial correlations in cell behaviour across a growing tissue.

We added the sentence clarifying this aspect:

“We assumed that molecules are homogeneously distributed within cells, and therefore, upon cell division, sister cells have the same concentration of ATML1 and Target variables at birth, but can have different cell sizes.”

This would be the most parsimonious assumption in cell content inheritance, which assumes cell contents are homogeneously distributed, and therefore, the concentration remains the same between sister cells.

We also had a former hypothesis that the inheritance of molecular correlations by daughter cells might lead to sister giant cells appearing in the tissue, and therefore driving spatial correlations across the tissue. This is a very interesting hypothesis that will need a much deeper study supported by much more time-lapse data, and it goes beyond the scope of this paper. However, in the discussion, we mention this hypothesis, which we now extend to explicitly mention the inheritance of molecular factors:

“Nevertheless, in a proliferating tissue, other mechanisms might operate at the same time that result in giant cell clustering. For instance, correlative effects on cells belonging to the same lineage might influence cell fate decisions, e.g., due to the inheritance of molecular factors from mother to daughter cells (40).”

How does spatial structure emerge? Looking at Fig 9B I can see some cells that have divided with a vertical (in the figure) division plane. What’s to stop this occuring more than once and pushing apart the lateral structure of the tissue? Why do the giant cells (and others) have thickness differences (tapering)? It seems like there’s a tremendous amount of detail here that is swept into “the multicellular tissue grows anisotropically” (L910).

We believe the key point to emphasize in this manuscript about how the spatial structure emerges is the fact of having giant cells, which do not divide, surrounded by dividing cells. Further details on the division planes will indeed determine other properties of the spatial structure of the overall pattern, but we believe this goes beyond the scope of this manuscript.

More details on the model can be found in Meyer et al. (2017). Yet, to clarify the growth rule, we added the following sentence:

“This anisotropic growth was implemented by imposing a displacement of the vertices with respect to the center of mass of the tissue, with a given radial and vertical exponential rate.”

This model does not take into account nor model cell wall thickness; the visualised thickness of the cell wall is an artifact of the visualization. We hope that the above

sentence is enough to clarify that there is no cell wall thickness affecting the growth nor any other mechanical effect in the growth, given that the growth was imposed.

In the methods, we mention that cells divide using the shortest path rule, together with the constraint of having the division plane through the center of mass of the cell. The vertical anisotropic growth makes it more likely that cells will divide with a horizontal division plane. However, after one such horizontal division, the cell shape changes, and if the next division occurs rapidly, the shortest wall might fall in the vertical direction. Such vertical divisions are also observed in the real growing sepal in live imaging data.
