# Supplementary material for: A common pathway controls cell size in the sepal and leaf epidermis leading to a nonrandom pattern of giant cells
Source: PLoS Biol. 2025 Nov 3;23(11):e3003469. doi: 10.1371/journal.pbio.3003469 (PMC12599956; doi:10.1371/journal.pbio.3003469)
Supplement: Attachment — Submitted filename: Response to reviewers 9-18-25.pdf [file pbio.3003469.s027.pdf]

We thank Iain Johnston for another round of thoughtful comments.

Reviewer #2, Iain Johnston (note, reviewer 2 has signed this review): The authors have input a tremendous amount of work into their edits and to my eyes things are substantially improved. My main points about the difficult layout are resolved, lots of individual questions have been answered, and I really appreciate the additional exploration and alternative approach in the randomisation questions I had earlier. The technical content (esp Figs. S18-19) has resolved my questions about the computational randomisation; the rhetorical changes have largely addressed my points about the interpretation, although it's on that point that I still have (minor) points to raise, as well as a few easy fixes below.

The authors have rephrased arguments about clustering; to respond to their question in the response letter I think "more clustered with respect to the rest of the tissue" would be a reasonable phrasing.
There remain in the manuscript a lot of mentions of "more than expected by chance". I think this discussion has highlighted the need to be more precise about this. "By chance" could mean "if every aspect of development was completely random" or "if cell identities in the final tissue were randomly assigned". The latter is the real result, but without reading the full paper I imagine most readers' instinct will be more along the lines of the former. I think that throughout (including l28 in the abstract), mentions of "by chance" should be replaced with a more precise statement e.g. "than expected from a randomised null model".

Thanks, we have revised to clarify “than expected from a randomised null model” throughout.  See below for more instances.

There also remain some implications that a "non-random mechanism" is responsible for the observed patterning. But I would question this (as I previously did) -- the "mechanism" is not directed, deterministic control, but rather an expansion of randomness in cells that aren't giant. It is not a "non-random mechanism", but a difference in the extent of randomness in giant vs other cells that is shaping the arrangement. I expand on this labelled ** below.

This was also revised as described below.

IJ

l120 -- "more than expected by chance" -> "more than expected by chance, reflecting the tissue's developmental history".
Edited as suggested.

l133 puzzle -> puzzle piece [the puzzle is usually a rectangle!]
Edited as suggested.

l188 -- this continuous value for total nuclear signal still feels strange. My question was less about whether signal is genuinely correlated with ploidy, and more about whether the obvious noise in the signal (any departure from tight integer peaks is a departure

from perfect readout of ploidy) challenges the interpretation. Perhaps not -- but I think it would feel more comfortable if the continuous and noisy nature of this readout was explicitly mentioned.

We have added: “For this reporter, the H2B-TFP signal distribution is noisy and continuous, not divided into four discrete peaks, providing an approximation of ploidy, not exact DNA content.”

l217 -- at the same proximal-distal position -> occupy the same proximal-distal arrangements [we're talking about the set of cells, right?]

We have edited to clarify further: “Large cells are observed in the same proximal–distal region on abaxial and adaxial sides during development (Fig. 3C–D). The region expands in the proximal direction as development progresses.”

l280 -- no difference -> no statistically significant difference

This has been corrected.

l282 -- this result feels awkwardly like cherry picking -- just for this mutant, one statistic (number per area) didn't give $p < 0.05$, so we look at another (fractional area of giants) which does. We're not normally allowed to do this, because there'll always be some statistic that gives $p < 0.05$ under the null hypothesis. Did the alternative statistic (fractional area of giants) also give $p < 0.05$ in the other mutants?

We have amended the test to explain we are testing another property to the system: “However, no statistically significant difference in the number of giant cells per unit area was observed between wild type and *ATML1-OX* (9 dpg: $p = 0.213$, 25 dpg: $p = 0.75$). Because the giant cells in *ATML1-OX* are so much bigger than wild-type giant cells, each of the giant cells in a given area of *ATML1-OX* cells takes up a large amount of space, resulting in few giant cells per unit area despite the fact that most of the unit area is occupied by giant cells. We sought to quantify what was apparent visually by comparing the fractional area occupied by giant cells between *ATML1-OX* and wild type and found that the fractional area occupied by giant cells was significantly higher in *ATML1-OX* (9 dpg: $p < 0.005$, 25 dpg: $p < 0.005$). Thus, in *ATML1-OX* the number of giant cells is not changed but the fractional area covered by giant cells is increased.”

l291 -- the entire cell-size distribution -> other aspects of the cell-size distribution
Edited as suggested.

l296 -- cell size distributions -> normalised cell size distributions
[note that there's some inconsistency in the use of "cell size" vs "cell-size"]
Note there are three Wasserstein Distance Plots. Only the third one uses the normalized cell size distribution. Therefore, only the third one is specified as normalized.

We have made it consistently cell-size when it is an adjective and cell size when it is a noun.

l333 -- double space after having

This has been corrected.

l394 -- I'd remove "slightly" -- I think they are dramatically affected! -- but best to just objectively report "affected". I'd pull the science bit of this paragraph "Several measures... formed a null distribution" into a new paragraph below the rest of this one, separating the model setup/validation and the scientific objective.

Slightly was removed. The paragraphs were split slightly differently than recommended to avoid continuity errors.

l1514 -- also remove "slightly"

Slightly was removed.

l401, l422, l426, l575 -- "than expected by chance" -> "than in a randomized null model"
We have changed these phrases as suggested.

**
l431 -- a non-random mechanism -- this is really my main question about the phrasing of the intepretation (as before). Is an expansion of external randomness around a quenched structure really a non-random mechanism? I don't think so -- to me a "non-random mechanism" implies some directed process, rather than quenched structure on a background of increasing entropy. It's actually explicitly the *expansion* of randomness that is inducing the signal of clustering. Perhaps it's safer to say "the giant cell arrangement must be subject to less random influence than the rest of the tissue" -- which is both true and reflects the actual mechanism that is revealed latter.

We chose to remove this sentence.

l454 -- "regulated biological mechanism" -- I think this is even more controversial. The preceding aspects of the paper have shown genetic regulation, but these simulation studies don't give direct support to the regulated nature of this "mechanism" governing arrangement. I would rephrase like the previous point, or just omit this -- the previous sections have already shown where regulation takes place.

We have removed this sentence.

l495 -- I'm not sure what the link to Fig. 7A should point to, but I don't think it should point to Fig. 7A

This has been corrected, it refers now to Fig. 5A.

l502 -- same with link to Fig. 4D?

This has been corrected, it refers now to Fig. 5D.

l550 -- "by chance" -- "under a random model where all final cells have random identities" -- or similar

Edited to read: “under a random model where all cells have random positions.”

l863 -- "such that the variances" -- but that's not the full story right? If you wanted to compare the variances you could just look at the absolute difference in variances as your MDS distance. Using Wasserstein means that you're also reporting all higher moments of the distributions too. That's fine, but this text shouldn't imply otherwise -- perhaps "such that all higher moments of the area distributions can be compared"

This edit has been made as suggested.

para l957 -- lots of Fig. 19 instead of Fig. S19

This has been corrected.
